# Hybrid bio-photo-electro-chemical cells for solar water splitting

**DOI:** 10.1038/ncomms12552

**Published:** 2016-08-23

**Authors:** Roy I. Pinhassi, Dan Kallmann, Gadiel Saper, Hen Dotan, Artyom Linkov, Asaf Kay, Varda Liveanu, Gadi Schuster, Noam Adir, Avner Rothschild

**Affiliations:** 1 The Nancy & Stephen Grand Technion Energy Program (GTEP), Technion—Israel Institute of Technology, Technion City, Haifa 32000, Israel; 2 Department of Materials Science and Engineering, Technion—Israel Institute of Technology, Technion City, Haifa 32000, Israel; 3 Schulich Faculty of Chemistry, Technion—Israel Institute of Technology, Technion City, Haifa 32000, Israel; 4 Faculty of Biology, Technion—Israel Institute of Technology, Technion City, Haifa 32000, Israel

## Abstract

Photoelectrochemical water splitting uses solar power to decompose water to hydrogen and oxygen. Here we show how the photocatalytic activity of thylakoid membranes leads to overall water splitting in a bio-photo-electro-chemical (BPEC) cell via a simple process. Thylakoids extracted from spinach are introduced into a BPEC cell containing buffer solution with ferricyanide. Upon solar-simulated illumination, water oxidation takes place and electrons are shuttled by the ferri/ferrocyanide redox couple from the thylakoids to a transparent electrode serving as the anode, yielding a photocurrent density of 0.5 mA cm^−2^. Hydrogen evolution occurs at the cathode at a bias as low as 0.8 V. A tandem cell comprising the BPEC cell and a Si photovoltaic module achieves overall water splitting with solar to hydrogen efficiency of 0.3%. These results demonstrate the promise of combining natural photosynthetic membranes and man-made photovoltaic cells in order to convert solar power into hydrogen fuel.

## Abstract (web summary)

Photoelectrochemical water splitting uses solar power to decompose water to hydrogen and oxygen. Here, the authors integrate thylakoid membranes extracted from spinach into a bio-photo-electro-chemical cell capable of overall water splitting without the need for any sacrificial reagents.

## Graphical abstract

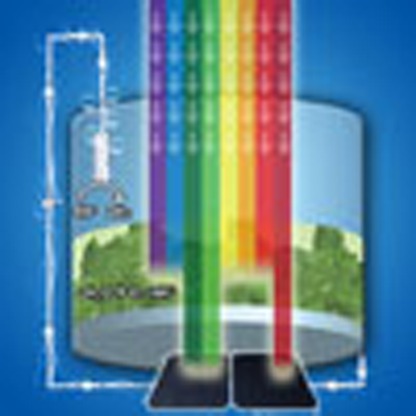

The need for cost effective and sustainable solutions for storing intermittent solar power has spurred a growing interest in artificial photosynthesis and solar fuels^1^,^2^,^3^. The most elementary solar fuel production process relies on the water–hydrogen cycle, 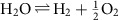. The forward reaction is work-consuming (endergonic). Under standard conditions it requires a minimum input of 237 kJ of entropy-free energy (that is, work) to dissociate one mole of water, which is the standard Gibbs free energy of the reaction (Δ*G*^0^). The reaction products, H_2_ and O_2_ gases, serve as fuel that can be stored in separate vessels and converted back into power by the reverse reaction. Thus, H_2_ can be produced sustainably by water electrolysis using renewable power sources such as solar power. Replacement of the entire worldwide primary energy consumption (12,910 million tons oil equivalent in 2014; ref. 4) by solar H_2_ produced at an average solar to hydrogen (STH) conversion efficiency of 1%, equivalent to the solar to biomass conversion efficiency of high efficiency crop plants^5^, would demand a net area of 9.7 million km^−2^ of solar collectors. This accounts for 6.5% of the total land area or 70.3% of the estimated arable land area on earth, a scenario which is not very practical^6^.

This limitation motivates research on other routes to store solar energy as chemical bonds in fuel products, for example through biotechnological or artificial photosynthesis processes. Bio-processes for renewable H_2_ production have been explored using fermentative and photosynthetic organisms as cellular factories for H_2_ production. Traditionally, these bio-processes are categorized into: (i) dark fermentation, (ii) photo-fermentation and (iii) biophotolysis^7^. Through the fermentative processes, protons serve as electron acceptors from catabolized organic compounds, while H_2_ production by biophotolytic processes make use of organisms capable of oxygenic photosynthesis, for example, green microalgae or cyanobacteria. For the latter, sunlight drives the oxidation of water molecules that serve as the electron donor, and protons generated are subsequently reduced to molecular hydrogen. A major barrier to commercialization of these technologies is the necessity to impose substantial restrictions on the bioreactor operating conditions in order to generate significant quantities of H_2_; H_2_ production with green algae occurs only under near anaerobic conditions, as the primary catalytic enzyme involved ((Fe–Fe)-hydrogenase) is inhibited by oxygen. Sustained H_2_ production using algae can be achieved only when photosynthetic O_2_ evolution is severely inhibited by sulfur deprivation, to such an extent that it equals the O_2_ consumption in the cellular respiration. Similarly, H_2_ production from filamentous or unicellular cyanobacteria requires the removal of O_2_ from the growth medium^8^.

The work described here puts forward a different approach that brings together light and photosynthetic membranes in a bio-photo-electro-chemical (BPEC) cell that generates, under illumination, an electromotive force that facilitates the water splitting reaction and funnels electrons and protons for the generation of H_2_. The operation of the BPEC cell requires the concerted operation of three components: (i) a photochemical module that captures light and produces separated charge carriers with excess chemical potential, (ii) a catalytic module that employs the photo-excited charge carriers to carry out endergonic oxidative or reductive chemistry, and (iii) an efficient method for transferring the charge carriers between the two modules while keeping as much of their excess free energy as possible^8^. In previous studies, photosystem I (PSI) complexes isolated from thermophilic cyanobacteria and tethered to either Pt nanoparticle catalyst^9^ or [NiFe]-hydrogenase^10^ were reported to produce up to 170 mol H_2_ (mol PSI min)^−1^, under illumination, in the presence of sacrificial electron donor (sodium ascorbate). McCormick *et al*.^11^ reported a BPEC cell that uses a two-step process to produce H_2_ at a rate of 2.4 μmol H_2_ (mg Chlh)^−1^, using live culture of the cyanobacteria *Synechocystis* PCC 6803, electron mediator (ferricyanide, Fe(III)CN), and a Pt cathode. Recently, Wang *et al*.^12^ reported the overall water splitting by self-assembled photosystem II (PSII) membranes on artificial catalysts, achieving H_2_ production rate of 9.1 μmol H_2_ (mg Chlh)^−1^. In this system, the PSII membranes were irreversibly attached to particles containing ruthenium or rhodium, along with other inorganic components, in the presence of Fe(III)CN. In another recent study, Mersch *et al*.^13^ presented a BPEC cell that couples PSII membranes and hydrogenase to mesoporous transparent electrodes, using 2,6-dichloro-1,4-benzoquinone (DCBQ) as an electron mediator. A benchmark water splitting photocurrent of 450 μA cm^−2^ was obtained at an applied bias of 0.9 V under red light illumination (*λ*=660 nm) at an intensity of 10 mW cm^−2^, corresponding to a light-to-hydrogen conversion efficiency of (1.5±0.1)%. 

Unlike conventional (first generation) biofuel production technologies from crop plants, a process that has been reported to work at an average efficiency of up to 1% and creates a serious competition for food^5^, BPEC cells make use of leaves, and are not limited to a particular plant species. Leaves of most crops do not have high commercial value, being mostly used as feedstock. We envision the utilization of crop leaves for green and benign H_2_ production, as an intermediate step after the crop plant has given its fruits, and before being fed to animals. Thus, in this work we present a BPEC cell that produces H_2_ gas using crude thylakoid membranes (henceforth called thylakoids) extracted from spinach, which is used here as a plant model. The thylakoids are extracted with minimal preparatory effort and then applied simply by letting them settle onto the surface of a transparent electrode, thereby enabling easy replacement of photodamaged thylakoids by fresh ones. Under illumination, a photocurrent is produced, mediated by Fe(III)CN, and channelled to evolve H_2_ at the cathode at applied bias far below the reversible voltage of water electrolysis (1.23 V in standard conditions). Furthermore, we present a stand-alone mode of operation, wherein the BPEC cell is coupled in tandem to a Si photovoltaic (PV) module that provides the bias required to produce H_2_. Thus, H_2_ is produced by this hybrid tandem cell from oxidized water molecules without any external power source or sacrificial electron donors.

## Results

### BPEC cell

**Figure 1:**
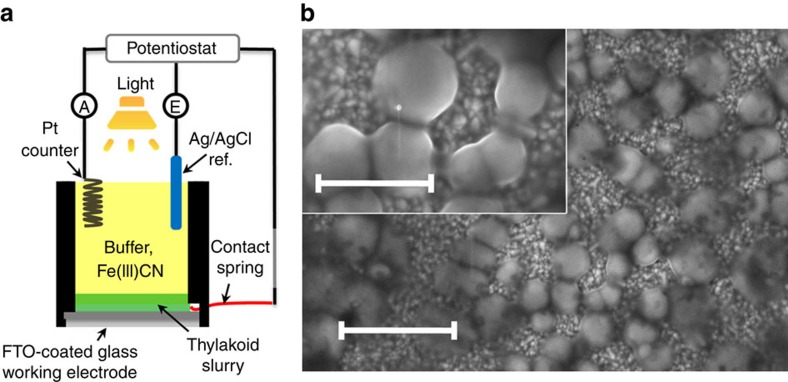
The BPEC cell. (**a**) Schematic illustration of the BPEC cell. The cell comprises a cylindrical container with a window at the bottom, where a transparent working electrode (FTO coated glass) serving as the anode is sealed against a rubber gasket. The anode is electrically connected through a contact spring to a potentiostat, which is set in three-electrode mode (except for the two-electrode measurements described towards the end of the article), along with an Ag/AgCl reference electrode and a Pt counter electrode (that is, cathode). The potentiostat measures the current between the working and counter electrodes using an ammeter {A}, and the potential {E} of the working electrode with respect to that of the reference electrode. A minimal amount of thylakoids settles from a slurry of crude spinach thylakoids in buffer solution containing glycerol onto the exposed area (1.08 cm^2^) of the FTO anode. The cell cavity is filled with 20 ml buffer A solution, containing the redox mediator Fe(III)CN (K_3_Fe(CN)_6_), which serves as a recyclable electron shuttle between the thylakoids and the anode. (**b**) Scanning electron microscopy image of the spinach membranes on the surface of a FTO coated glass electrode. Small amount of membranes were placed on the electrode and the scanning electron microscopy images were taken in vacuum. Scale bar, 2 μm. Inset: scanning electron microscopy image at higher magnification. Scale bar, 1 μm.

Our BPEC cell design meets the following principles: (i) Utilization of crude thylakoids without chemical immobilization or modification. This prevents the deleterious outcomes of immobilization procedures to the photosystems^14^, and therefore, it is expected to retain high photosynthetic activity, as well as enable easy replacement of photodamaged thylakoids; (ii) Utilization of a durable and inexpensive transparent electrode; and (iii) Use of a stable electron mediator (Fe(III)CN) to allow efficient electron extraction from the thylakoids and fast charge transfer to the anode^11^,^12^. The use of crude thylakoids purified from spinach in a BPEC cell is schematically illustrated in Fig. 1a. The anode is a transparent electrode made of fluorinated-tin-oxide (FTO) coated glass substrate onto which the thylakoids were applied simply by letting them to settle from a slurry of crude spinach thylakoids in buffer solution onto the anode. Figure 1b shows scanning electron microscopy images of the thylakoids on the FTO surface. For clarity of observation, only a small amount of thylakoids was used in the preparation of the scanning electron microscopy specimen. The thylakoids were not tightly attached to the anode, and could easily be washed away by gently flushing with buffer solution. Thus, stirring of the thylakoids during operation of the BPEC cell was impossible and not deemed critical for performance.

### Significant photocurrent from spinach thylakoids

**Figure 2:**
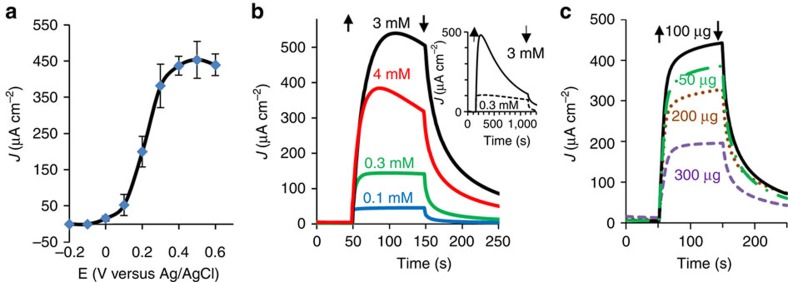
Photocurrent measurements in three-electrode mode. (**a**) Photocurrent density as a function of the electrode potential, measured in buffer A solution with a thylakoid content of 0.1 mg Chl and Fe(III)CN concentration of 3 mM. The error bars represent the s.d. over four independent measurements. (**b**) The photocurrent, measured at an electrode potential of 0.5 V_Ag/AgCl_, as a function of time during exposure to solar-simulated light between *t*=50 and 150 s. Four measurements in buffer A solution with a thylakoid content of 0.1 mg Chl and Fe(III)CN concentrations of 0.1, 0.3, 3 and 4 mM are presented (blue, green, black and red curves, respectively). The inset shows the long-term photocurrent stability in high (3 mM, full line) and low (0.3 mM, dashed line) Fe(III)CN concentrations. The arrows indicate light turn on (up) and off (down) points. (**c**) The photocurrent dependence on the amount of thylakoids (expressed in mg Chl) in solution. Maximal photocurrent was obtained with 100 μg Chl (black line), whereas 50, 200 or 300 μg Chl (green broken, brown dotted or purple dashed lines, respectively) yielded lower currents. The measurements were carried out in buffer A solution with 3 mM Fe(III)CN.

Under solar-simulated illumination, water oxidation is catalysed by the PSII complex embedded in the thylakoids, releasing O_2_ and protons. A mediated photocurrent is obtained in the system by use of the redox couple ferri/ferrocyanide (Fe(III)/Fe(II)CN) that extracts electrons from the photosynthetic membranes and transfers them to the anode. The photocurrent dependence on the applied potential is presented in Fig. 2a, showing saturation of 450±50 μA cm^−2^ at an anode potential of 0.5 V_Ag/AgCl_. The measurements were carried out in buffer A solution with a thylakoid content of 0.1 mg Chl and a Fe(III)CN concentration of 3 mM. The dependencies of the photocurrent, measured at 0.5 V_Ag/AgCl_, on the Fe(III)CN concentration (at a thylakoid content of 0.1 mg Chl) and thylakoid content (at a Fe(III)CN concentration of 3 mM) are presented in Fig. 2b,c, respectively. A maximal photocurrent density of *ca.* 0.5 mA cm^−2^ was obtained using 3 mM Fe(III)CN and thylakoid content of 0.1 mg Chl.

Previous reports have shown photo-induced electron transfer from PSII and PSI^15^ and the production of photocurrent from spinach thylakoids utilizing Fe(III)CN as electron shuttle, as well as using other thylakoid sources, different types of electron mediators, and a variety of electrode compositions. For example, Calkins *et al*.^16^ immobilized spinach thylakoids onto multi-walled carbon nanotubes, and achieved a maximal photocurrent of 68 μA using Fe(III)CN as the electron mediator. We have previously reported a photocurrent density of 100 μA cm^−2^ using a graphite electrode and DCBQ as an electron shuttle^17^. Hasan *et al*.^18^ reported a maximal photocurrent density of 130 μA cm^−2^ using a gold electrode and *para*-benzoquinone electron transfer mediator. Thylakoids have also been used without an electron mediator^19^,^20^, though the reported photocurrents were lower compared with reports with an electron mediator. Larom *et al*.^21^ reported the production of an unmediated photocurrent density of 16 μA cm^−2^ using crude *Synechocystis* membranes and N-acetyl cysteine-modified gold electrode. A photocurrent density of 42 μA cm^−2^ was reported by Hamidi *et al*.^22^, using immobilized spinach thylakoids in osmium polymer network on a graphite electrode. Other studies reported high photocurrents using purified PSI or PSII. A solid state electrochemical device^23^ that combines purified photosynthetic complexes with a transparent electrode coated with a sandwich of metallic nanolayers reached a photocurrent density of 120 μA cm^−2^. This study is mentioned in a recent review article on photosynthetic protein based PV devices^24^ as setting the record for these devices. Isolated PSII membranes were also employed by Mersch *et al*.^13^, reporting photocurrent densities as high as 930 μA cm^−2^ at an applied potential of 0.5 V versus the normal hydrogen electrode (NHE). However, the photocurrent in this study was measured under red light illumination (*λ*=679 nm, 10 mW cm^−2^), which makes it difficult to compare with our results that were measured under solar-simulated white light illumination. Hence, the photocurrent density reported here sets a new record for solar-simulated measurements of BPEC cells utilizing any photosynthetic membranes, crude or purified ones. Furthermore, the preparation of isolated reaction centers is expensive, time and energy consuming and requires the use of polluting detergents, and the photocurrent quickly decays due to irreversible degradation of the reaction centers^13^,^16^,^18^,^19^,^22^. Our approach alleviates these drawbacks by using crude thylakoids that can be readily replaced by fresh ones, as demonstrated below.

To estimate the efficiency of the charge transfer from the thylakoids to the electric circuit we calculated the ratio between the total photo-induced charge, *Q*_photo_, measured by integrating the photocurrent (*I*_photo_) over time, 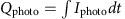, and the amount of O_2_ that evolved by the photosynthetic membranes, ΔO_2_ (measured in (mol O_2_)). Thus, the charge transfer efficiency (*η*_ct_) was calculated according to equation (1):


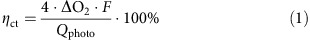


where *F* is the Faraday constant. In order to calculate ΔO_2_ we measured the increase in oxygen concentration after 10 min of illumination using a Clark electrode (see Supplementary Fig. 1a). The charge transfer efficiency was (94±11)% (Supplementary Fig. 1b). This value indicates that under these conditions almost all of the electrons derived from water photo-oxidation were successfully transferred to the electric circuit.

### Photoactivity lifetime

Fe(III)CN reduction by isolated chloroplasts or sub-chloroplast components is one of the early facilitators of the Hill reaction and has been known since the 1950's. Studies have suggested that Fe(III)CN is reduced by several separate donor sites located in PSII, plastoquinone (PQ) and the cytochrome *b*_*6*_*f* or PSI^25^. Earlier studies have made clear that the reduction is accompanied by an adverse effect which rapidly inhibits photosynthesis. A dose-dependent perturbation of 20–50% in the oxygen evolution rate was reported for spinach chloroplasts that were dark-incubated in 0.5–20 mM Fe(III)CN for 20 min^26^. Furthermore, Kirilovsky *et al*.^27^ investigated the complex light-dependent PSII inactivation caused by Fe(III)CN incubation by electron paramagnetic resonance (EPR) spectroscopy and fluorescence induction techniques. Their report suggests that the inhibition is the result of a dual effect; the first one is the over-oxidation of the donor site in PSII, which uncouples the oxygen evolving center, and the second one is the formation of abnormal states of reduced *Q*_A_ at the acceptor site.

**Figure 3:**
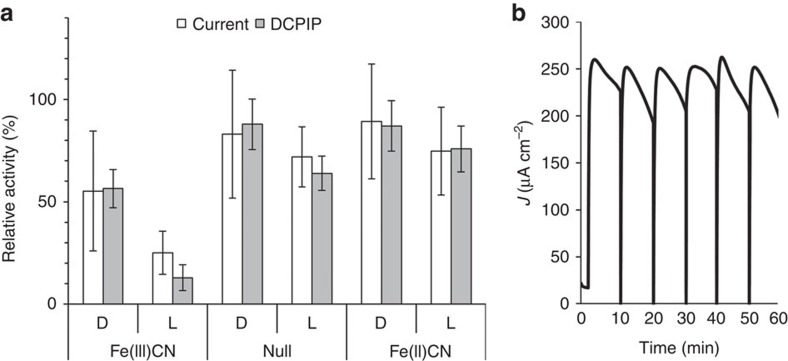
Light-induced reduction of Fe(III)CN damages the photosynthetic activity of the thylakoids. (**a**) The residual photocurrent and DCPIP reduction obtained from thylakoids after incubation for 10 min in the dark (D) or in light (L), without (null) or with 3 mM Fe(III)CN or Fe(II)CN, normalized to a control experiment using identical thylakoids without incubation. The error bars represent the s.d. over five measurements. (**b**) Batch mode of operation wherein damaged thylakoids were replaced with fresh ones every 10 min. All the other cell components were reused.

Here we study a system that exploits Fe(III)CN as an electron mediator from photo-activated thylakoids. As expected, photocurrent degradation was observed, especially at high photocurrent densities (see inset of Fig. 2b). Thus, we sought to elucidate the nature of the photocurrent degradation. Towards this end, spinach thylakoids were placed in the BPEC cell and incubated in light or dark in the presence or absence of ferricyanide (Fe(III)CN) or ferrocyanide (Fe(II)CN) as a control, and then used, subsequently, for photocurrent and photosynthetic electron flow measurements. Figure 3a presents the residual photocurrent that was measured following 10 min of different incubation treatments. The residual activities are expressed as the ratio between the photocurrent and PSII activities obtained after the incubation to those obtained in a control experiment using identical thylakoids without 10 min of incubation. Incubations in light or dark without Fe(III)CN were found to reduce the photocurrent to about 60 and 80% of the initial photocurrent, respectively, indicating that light enhances the degradation of the photocurrent. In addition, there is significantly more reduction of the photocurrent when the thylakoids are incubated with Fe(III)CN in the light, resulting with only 20% of the initial photocurrent (Fig. 3a). To rule out the possibility that the reduced form of the electrolyte, that is Fe(II)CN obtained from the reduction of Fe(III)CN in the light, degraded the photocurrent, thylakoids were also incubated with Fe(II)CN under the same conditions. The degradations that were observed following incubation with Fe(II)CN in the dark or light were comparable to that of thylakoids that were incubated without Fe(III)CN (Fig. 3a). This observation is consistent with reports that Fe(II)CN ions are harmless to chloroplast or sub-chloroplast components in the mM concentration range^28^.

Since the BPEC cell makes use of only a small amount of thylakoids in the presence of excess Fe(III)CN in solution, we hypothesized that the light-dependent photocurrent decay may have occurred as a result of the PSII membranes reaching their turnover number limit, after which PSII was damaged. To examine this hypothesis and to determine the fraction of photosynthetically active electron flow in the thylakoids in each of the incubation conditions, thylakoids were collected from the BPEC cell after 10 min of incubation, the Fe(III)CN was washed out by centrifugation, the thylakoids were re-suspended in fresh medium (as explained in the experimental procedures), and analysed for 2,6-dichlorophenolindophenol (DCPIP) reduction. The results are presented in Fig. 3a. There is correlation between the photocurrent degradation and the DCPIP reduction degradation. A residual photosynthetic electron flow activity of 10% was observed for thylakoids that were incubated with Fe(III)CN in light, compared with 60% of this activity that was retained in thylakoids that were incubated in light without Fe(III)CN. This observation suggests that the combination of light and Fe(III)CN is most harmful to the thylakoids, enhancing the photoinhibition of the photosynthetic electron flow. Thus, we conclude that the photocurrent degradation was caused in part by the photoinhibition deactivation of photosynthetic electron flow which is enhanced in the present of Fe(III)CN. Additional contribution of the Fe(III)CN to the degradation of the photocurrent and the photosynthetic electron flow occurred in the dark (Fig. 3a). In order to overcome the degradation problem, the thylakoids were replaced with fresh ones every 10 min in a time course of 1 h, while continuously monitoring the photocurrent. As shown in Fig. 3b, the photocurrent was kept at a high level throughout the entire hour, except for the intermittent exchange points every 10 min. This demonstrates that the electrodes and mediator solution may be reused for a long time.

### The photocurrent is derived from photosynthetic electron transfer

**Figure 4:**
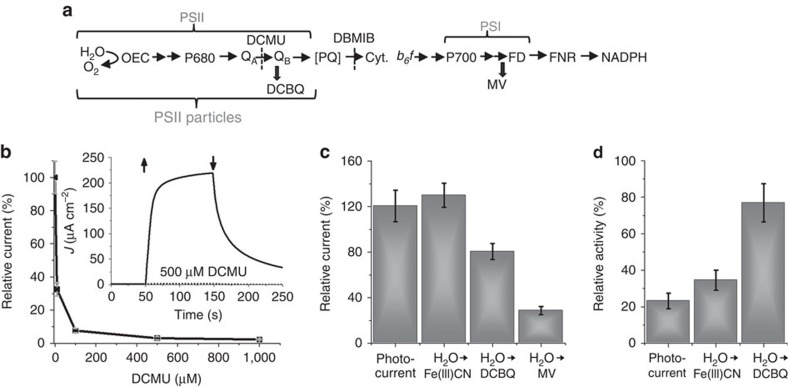
The photosynthetic electrons are extracted by Fe(III)CN following the *Q*_B_ site. (**a**) A scheme of the photosynthetic electron flow (horizontal arrows) with exogenous electron acceptors (vertical arrows) and electron flow inhibitors (vertical dashed lines). Double arrows mark additional electron acceptors. (**b**) Relative photocurrent of thylakoids, measured at a potential of 0.5 V_Ag/AgCl_ with 3 mM Fe(III)CN, as a function of DCMU concentration, averaged over at least three experiments. The relative photocurrent is the photocurrent measured with DCMU divided by the photocurrent measured without it, as shown in the inset. (**c**) Relative photoactivity measured with DBMIB (5 μM) with respect to measurements without DBMIB. The columns show (left to right) the relative photocurrent and oxygen evolution rates measured with Fe(III)CN or DCBQ as acceptors and the oxygen consumption rate using MV as electron acceptor. The results error bars represent the s.d. over at least three measurements. (**d**) Relative photocurrent and photoactivity of Fe(III)CN and DCBQ reduction rates in PSII particles, relative to the respective measurements with intact thylakoids (both at the equivalent amount of 0.1 mg Chl). It is noted that when measured at the equivalent amount of lower chlorophyll concentration such as 0.01 mg Chl, the H_2_O to DCBQ activity of the PSII particles was higher than that of intact thylakoids. The error bars represent the s.d. over at least three measurements.

To examine whether the source of electrons was indeed photosynthetic water photolysis and not spurious side reactions, the photosynthesis inhibitor 3-(3,4-dichlorophenyl)-1,1-dimethylurea (DCMU) was introduced to the system as a probe. DCMU is known to effectively block the electron transfer from *Q*_A_ to *Q*_B_ in PSII^29^,^30^, as illustrated in Fig. 4a (see also Supplementary Fig. 2). Hence, it inhibits continuous water photo-oxidation. Figure 4b shows the relative photocurrent with and without DCMU as a function of DCMU concentration. It is evident that an inhibitory effect governs the process, with a complete photocurrent arrest at 500 μM DCMU. The inhibition by DCMU implies that the source of electrons reducing the Fe(III)CN mediator must be at or down-stream the *Q*_B_ site, which is the site blocked by the herbicide^30^ (see Fig. 4a). Unlike the quinone acceptors DCBQ and DCPIP which accept electrons mostly from the quinone-binding sites of PSII (*Q*_B_) and Cytochrome *b*_*6*_*f* (cyt *b*_*6*_*f*), Fe(III)CN can accept electrons from several electron donor sites under different conditions, including *Q*_A_^30^, the PQ pool^31^, cyt *b*_*6*_*f* and PSI^32^. To further investigate the electron source of the reduction of Fe(III)CN in our system, we performed a series of experiments with the photosynthesis inhibitor 2,5-dibromo-6-isopropyl-3-methyl-1,4-benzoquinone (DBMIB). At low concentrations DBMIB blocks the electron transfer from the PQ pool to cyt *b*_*6*_*f* (refs 30, 33). This inhibition was observed in our system, as shown by the persistence of electron transfer from H_2_O to DCBQ but not from H_2_O to methyl viologen dichloride (MV) in the presence of 5 μM DBMIB, see Fig. 4c. DBMIB did not inhibit the photocurrent, as well as the H_2_O to Fe(III)CN electron transfer rate, indicating that Fe(III)CN extracts electrons before cyt *b*_*6*_*f*. Together with the observation that DCMU suppressed the photocurrent (Fig. 4b), namely, it blocked the Fe(III)CN reduction, the observation that DBMIB did not inhibit these photoactivities (Fig. 4c) implied that the first site to reduce Fe(III)CN along the electron transfer chain must be the PQ pool and/or the reduction of cyt *b*_*6*_*f* site (see Fig. 4a). Interestingly, the photocurrent increased in the presence of 5 μM DBMIB (Fig. 4c), indicating that electrons were extracted from PQ at a faster rate than from all the sites without the inhibitor, as reported elsewhere^30^,^32^.

Additional support to this conclusion was obtained by comparing the photoactivities of PSII particles prepared by the Berthold, Babcock and Yoccum (BBY) method (ref. 34) with those of crude thylakoids, see Fig. 4d. The photocurrent and H_2_O to Fe(III)CN electron transfer rate of the PSII particles were much lower than the respective values obtained with intact thylakoids (see Supplementary Fig. 3), despite the fact that PSII particles gave rise to higher specific activity when measured at a lower chlorophyll concentration than what was used here (see note in the legend of Fig. 4d). The PSII particles are enriched in PSII but contain less PQ and negligible amounts of cyt *b*_*6*_*f* and PSI^34^, compared with the thylakoids^35^. An additional explanation to the reduced photocurrent produced by the PSII particles as compared with that produced by intact thylakoids takes into account the reduced amount of PQ pool, which is about 3-fold lower in the PSII particles than in crude thylakoids^35^. The solution reduction potential is controlled by the concentrations of Fe(III)CN and Fe(II)CN, and is in equilibrium with the redox poise of the PQ pool, which is smaller and its turnover rate is slower in the PSII particles. Therefore, if PQ is reducing the exogenous Fe(III)CN, its reduced amount and turnover rate in the PSII particles results in a smaller photocurrent, as shown by the measurement of the H_2_O to Fe(III)CN electron transfer rate in Fig. 4d. Evidences for the beneficial contribution of PSI and cyt *b*_*6*_*f* complex to the photocurrent were reported by Calkins *et al*.^16^ and by Rasmussen and Minteer^36^ in similar systems. In the latter work, it was found that the dominant electron donor was PSI, followed by PSII and the cyt *b*_*6*_*f* complex^36^. In order to analyse whether or not Fe(III)CN is reduced by PSI in the present system, MV, which is a competitor to ferredoxin for accepting electrons from the Fa/Fb site of PSI^16^, was added to the system resulting with the reduction of the photocurrent by about 30% (see Supplementary Fig. 4). Altogether, the results in Fig. 4 show that the photocurrent was inhibited by DCMU but not by DBMIB, and that the PSII particles produced lower photocurrent than intact thylakoids, as well as the partial inhibition by MV, indicating that the mediator Fe(III)CN extracts photosynthetic electrons mostly from PQ, and possibly also from cyt *b*_*6*_*f* and PSI (to a lesser extent).

**Figure 5:**
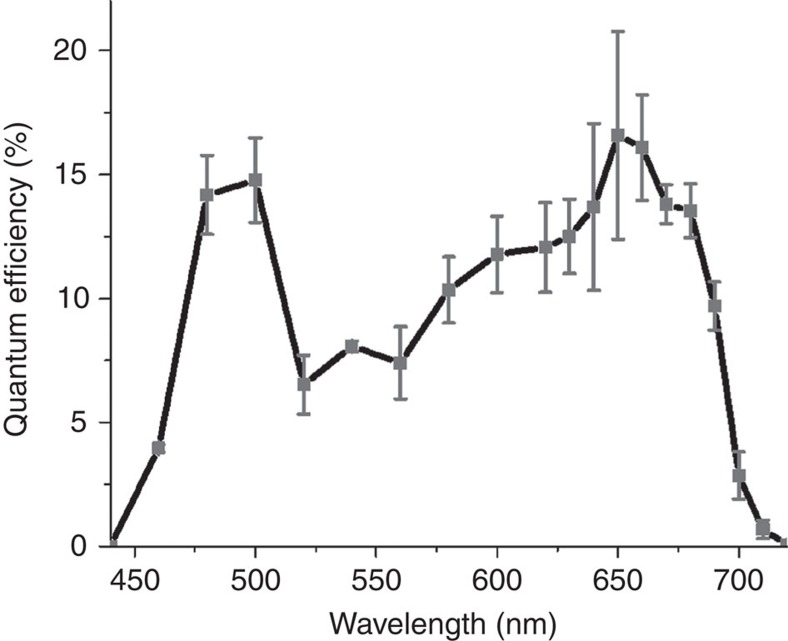
Quantum efficiency. The quantum efficiency, that is, the photo induced electrons per photons, measured at various wavelengths from 440 to 720 nm with a spectral bandwidth of 10 nm. Measurements were taken with different samples of thylakoids (0.1 mg Chl) for each wavelength. The photocurrent was measured at a potential of 0.5 V_Ag/AgCl_. The values error bars represent the s.d. over three measurements.

Further evidence to the involvement of photosynthesis in the photoactivity was obtained by analysing the spectral response of the photocurrent. We hypothesized that the photocurrent would be proportional to the light intensity absorbed by chlorophyll, the dominant pigment in plant thylakoids. Thus, we anticipated the maximal photocurrent to be obtained at wavelengths of 460 and 660 nm, where the light absorption of chlorophyll is maximal (see Supplementary Fig. 5). In order to examine this hypothesis, we measured the external quantum efficiency (EQE) spectrum using thylakoids in the amount equivalent to 100 μg Chl placed in the cell. The results are shown in Fig. 5, displaying two peaks at the same wavelengths as the peaks in the absorption spectrum of the spinach thylakoids (Supplementary Fig. 5). A maximum EQE of (17±4)% was obtained at 650–660 nm. A similar value was reported by Mersch *et al*.^13^, using mesoporous transparent electrodes with a much larger amount of PSII than we used here.

### Low bias hydrogen generation

**Figure 6:**
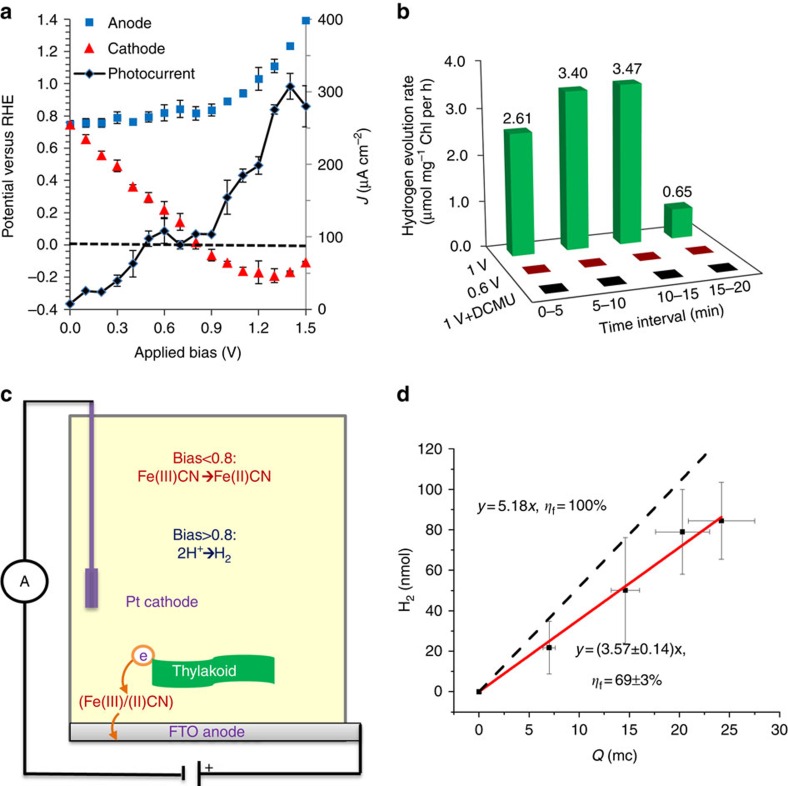
Two-electrode mode measurements. (**a**) Photocurrent (black curve) and anode (blue squares) and cathode (red triangles) potentials as a function of the applied bias between the anode and cathode. The dashed line represents a potential of 0 V_RHE_. The photocurrent was measured under exposure to solar-simulated light in a cell containing buffer A solution with a thylakoid content of 0.1 mg Chl and Fe(III)CN concentration of 3 mM. The error bars represent the s.d. over three independent measurements (**b**) Hydrogen evolution rate at the cathode under a bias of 0.6 V (red bars), 1.0 V (green bars) and 1.0 V with the addition of 0.5 mM DCMU. (**c**) Schematic illustration of the proposed electron transfer pathway in the BPEC cell. When the applied bias is lower than 0.8 V the cathode reaction is dominated by cyclic electron transfer of the Fe(III)CN/Fe(II)CN couple, whereas above 0.8 V proton reduction to hydrogen prevails. (**d**) H_2_ production as a function of the charge that was transferred between the anode and cathode. Dashed black line corresponds to a Faradic efficiency of 100%. The error bars represent the s.d. over four independent measurements.

Photoelectrochemical water splitting has been proposed as a promising strategy for capturing and storing solar energy in the form of chemical energy, for example hydrogen fuel^37^. Towards this end, we sought the conditions in which the photocurrent could give rise to the reduction of protons in order to form H_2_ at the cathode. Thus, the anode and cathode were connected to the potentiostat in two-electrode mode of operation, and the photocurrent was measured as a function of the voltage between the two electrodes. In addition, the potential of the cathode was monitored simultaneously by measuring the voltage drop between the cathode and a reference electrode, as illustrated in Supplementary Fig. 6a. This enabled simultaneous measurement of the photocurrent as function of the applied voltage between the anode and cathode, as well as the potentials of the two electrodes. The results are shown in Fig. 6a. For hydrogen evolution, the cathode must be at a potential lower than 0 V with respect to the reversible hydrogen electrode (RHE) scale. This condition is reached at a minimum voltage of 0.8 V (see Fig. 6a), where the onset of the water photoelectrolysis reaction takes place. In a control experiment using the same setup without the thylakoids, a voltage of 2 V was required to initiate the water electrolysis reaction (see cyclic voltammogram in Supplementary Fig. 6b). Furthermore, in the experiment without the thylakoids the potential of the cathode was higher than 0 V_RHE_ in the entire bias range of 0–1.5 V (see Supplementary Fig. 6c), indicating that no hydrogen evolution occurred in this range. Thus, the introduction of thylakoids to the BPEC cell reduced the minimum bias for the onset of the water splitting reaction from 2 to 0.8 V. The onset at 0.8 V is equal to the difference between the redox potential of the Fe(III) /Fe(II)CN redox couple and the hydrogen evolution reaction (HER) potential at a Pt electrode. This indicates that the light reactions split water and reduce Fe(III)CN, thus the Fe(III)/Fe(II)CN redox couple sets the anode potential, whereas the cathode potential reaches the HER potential at the onset of hydrogen evolution, giving rise to a bias of 0.8 V at the onset of water splitting with hydrogen evolution at the cathode. It is noteworthy that some photocurrent was measured below the onset of the water splitting reaction (see Fig. 6a). This photocurrent arose from other electrochemical side reactions, most likely the redox cycle of the Fe(III) /Fe(II)CN couple. The photocurrent associated with these processes saturated at ∼100 μA cm^−2^ at a bias of 0.5 V (see Fig. 6a). Between 0.5 and 0.8 V the photocurrent was constant, starting to increase again above 0.8 V and reaching ∼300 μA cm^−2^ at 1.4 V. Thus, the net water photoelectrolysis current density reached approximately 200 (=300–100) μA cm^−2^ at 1.4 V, indicating that the Faradaic efficiency for the water splitting reaction was *ca.* 67%.

In order to confirm this indirect estimation, the gases that evolved at the cathode headspace were collected and analysed by gas chromatography (GC). The H_2_ evolution rate at several time intervals during GC measurements at a bias of 0.6 or 1.0 V are presented in Fig. 6b. The H_2_ evolution rate between 5 and 15 min was found to be 3.4−3.5 μmol (mg Chl h)^−1^, being among the highest reported values for BPEC devices that operate without sacrificial electron donors. In control experiments, under a bias of 1 V and in the presence of 0.5 mM DCMU, or in the presence of Fe(III)CN but without thylakoids, H_2_ production could not be detected at all (Fig. 6b and Supplementary Fig. 6c), demonstrating that electron flow down-stream of PSII is crucial for H_2_ production. These observations are summarized in a schematic illustration of the proposed electron transfer pathways in the cell as depicted in Fig. 6c. Finally, the Faradaic efficiency (*η*_F_) is calculated from the ratio between the slops of the H_2_ versus Q (Fig. 6d), indicating that the total charge that transfers between the working and counter electrodes produces hydrogen at a (69±3)% efficiency. This value is consistent with the dual electron pathway suggested in Fig. 6c, wherein 2/3 of the photocurrent supports proton reduction at the cathode, whereas the remaining 1/3 feeds the Fe(III)/Fe(II)CN electron transfer cycle.

### Hybrid BPEC–PV tandem cell for solar water splitting

**Figure 7:**
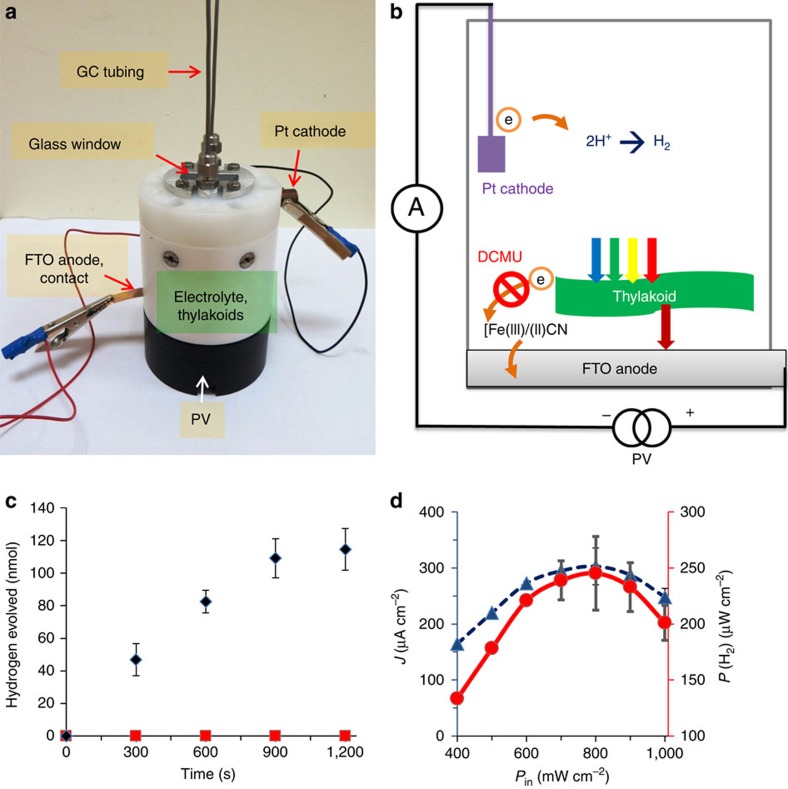
PV–BPEC tandem cell. (**a**) Photograph of the tandem cell. (**b**) Illustration of the electron transfer pathway in the tandem cell. (**c**) Hydrogen evolution at the cathode in the presence (red squares) or absence (black diamonds) of the herbicide DCMU. (**d**) The influence of the incident light intensity (*P*_in_) on the photocurrent (blue curve) and power accumulated in hydrogen bonds (red curve) produced by the tandem cell. The error bars in **c**,**d** represent the s.d. in four independent measurements.

The photo-activated spinach thylakoids in the BPEC cell significantly reduced the voltage needed to produce H_2_ from water. Nonetheless, an external bias of at least 0.8 V was still needed to energize the process. Aiming to construct a stand-alone solar-powered device that does not require an external power source to operate, the BPEC cell was coupled in tandem with a Si PV cell (see photograph in Fig. 7a) that provides the bias needed for the water splitting reaction, as illustrated in Fig. 7b. This design mimics the Z-scheme in photosynthesis, wherein PSI reenergizes the electrons from PSII. In the BPEC–PV tandem cell, water oxidation is energized by the short wavelength radiation of the sunlight spectrum, harvested by the thylakoids. In addition, the long wavelength radiation transmitted through the thylakoids is captured by the PV cell, activating it to produce the power needed to support the overall water splitting reaction. In other words, the PV cell replaces the potentiostat that was used in the experiments described in the previous sections. Such a tandem design has two important merits in regards to solar water splitting. First, coupling the cells in series to increase the photovoltage produced by the device enables overall water splitting, which neither one of the cells could drive alone. Second, unlike a single absorber system, where only photons possessing sufficient energy to drive the water photolysis reaction are used to stimulate the PV cell, in the tandem cell less energy is needed from each photon. This allows a larger wavelength threshold (that is, more efficient light harvesting) and smaller thermalization loss, thereby increasing the power conversion efficiency of the system^38^,^39^. To evaluate the performance of the hybrid BPEC–PV tandem cell, the gases produced in the BPEC cell were analysed by GC (Fig. 7c). H_2_ gas formation was observed only in the presence of all the components, namely the electrolyte solution, thylakoids and PV cell. Maximal H_2_ production rate was obtained within the first 15 min of operation, after which the rate decreased and finally plateaued after 20 min. These observations are consistent with the results obtained for the BPEC cell alone (see inset of Fig. 2b and Fig. 6b). Expectedly, the addition of the herbicide DCMU totally inhibited the H_2_ evolution (see Fig. 7c).

Last, the effect of light intensity on the photocurrent produced by the BPEC–PV tandem cell was examined. The power output of the cell was calculated according to equation (2):


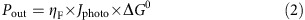


where *η*_F_ is the faradaic efficiency for the water splitting reaction (69%, see Fig. 6d), *J*_photo_ is the photocurrent density and Δ*G*^0^ is the chemical potential of the water splitting reaction (1.23 V per electron in standard conditions). The maximal photocurrent density and power output of the cell were obtained under illumination of 80 mW cm^−2^ (see Fig. 7d), yielding *J*_photo_=300±25 μA cm^−2^ and *P*_out_=250±20 μW cm^−2^. This corresponds to STH conversion efficiency, *η*_STH_=*P*_out_/*P*_in_ where *P*_in_ is the power density of the incident light, of (0.31±0.03)%. It is noteworthy that the hybrid BPEC–PV tandem cell reached maximum performance at 80 mW cm^−2^, less than full 1 Sun conditions (100 mW cm^−2^). The decrease in photocurrent at light intensities above 80 mW cm^−2^ (see Fig. 7d) is most likely due to photo-saturation of the thylakoids at this light intensity threshold.

## Discussion

We presented here a BPEC cell comprising of plant thylakoids in electrolyte solution that produced photocurrents as high as 0.5 mA cm^−2^ derived from photosynthetically catalysed water oxidation. Powering the cell with additional bias greater than 0.8 V leads to overall water splitting with hydrogen and oxygen production at a Faradic efficiency of (69±3)%. A stand-alone tandem cell comprising the BPEC cell and a Si PV cell was demonstrated, reaching STH conversion efficiency of 0.3%. This innovative hybrid system brings together natural photosynthetic membranes and man-made PV technology in order to solve one of the greatest challenges in renewable energy development, that is solar energy conversion and storage in hydrogen fuel. Further development of this promising approach should aim to enhance the STH conversion efficiency, for example, by replacing flat transparent electrodes with porous ones.

## Methods

### Plant material and thylakoid membrane isolation

Spinach (*Spinacia oleracea*) grown in fields of the lower Galilee between the coordinates 33^o^4′18.4″N to 32^o^51′50.5″N and 35^o^16′32.8″E to 35^o^6′24.2″E was obtained from the local growers. The results presented in this work were obtained using three independent biological preparations. The thylakoid membranes preparation was carried out by grinding the young leaves in buffer A (50 mM 2-(N-morpholino)ethanesulfonic acid (MES)/NaOH pH=6.0, 15 mM NaCl, 5 mM MgCl, 2 mM CaCl_2_), and precipitating the thylakoids according to the procedure described by Andreasson *et al*.^40^ PSI I enriched membrane fragments (BBY membranes) were extracted using the detergent Triton X-100 (Sigma), as described by Berthold *et al*.^34^ The chlorophyll content in thylakoid preparations was determined according to the procedure described by Arnon^41^.

### Photoelectrochemical measurements

The photocurrent produced by the thylakoids was measured in the BPEC cell illustrated in Fig. 1a. The measurements were carried out at ambient temperature (25±2 °C). 0.1 mg Chl *a* of the thylakoids in buffer A solution containing 10% v/v glycerol were set on a FTO-coated glass electrode placed at the bottom of the BPEC cell. The BPEC cell was illuminated from the top by a solar simulator (Oriel Sol3A class AAA solar simulator, Newport, USA) calibrated to provide solar simulated (AM1.5G) light intensity of 1 Sun (100 mW cm^−2^) at the BPEC cell. The operating conditions were chosen based on a series of cyclic voltammetry measurements, and carried out using a potentiostat (Zennium, ZAHNER-elektrik, Germany) connected either in a three-electrode mode with an Ag/AgCl (in 3 M NaCl solution) reference electrode (RE-1B, CH Instruments, USA) and a platinum wire counter electrode, or in a two-electrode mode to a platinum wire counter electrode. Fe(III)CN mediated photocurrents were measured chrono-amperometrically at different potentials in buffer A solutions with different amounts of potassium ferricyanide (K_3_Fe(CN)_6_, Sigma Aldrich) as described in the text, under cyclic exposure to light–dark pulses. In order to examine the electron source, the herbicides DCMU (Fluka) and DBMIB (Aldrich) were used. The photocurrent density was calculated as the quotient of the current under illumination minus the dark current, divided by the projected area of the electrode (1.08 cm^2^) that was exposed to the light and in direct contact with the solution containing the thylakoids (Fig. 1a). The standalone solar water splitting tests were carried out by coupling the BPEC cell in tandem with a Si PV module (IXOLAR XOB17-04x3 SolarBit, IXYS, Korea). The photocurrent between the working (TEC15) and counter (Pt wire) electrodes and the voltage drop between the working and reference (Ag/AgCl) electrodes were monitored by digital multimeters (34401A DMM, Agilent Technologies, USA) and recorded digitally using LabVIEW (National Instruments, USA).

### Quantum efficiency measurements

The EQE of the BPEC cell with 0.1 mg Chl and 3 mM Fe(III)CN was measured at a potential of 0.5 V_Ag/AgCl_. The thylakoids were illuminated by monochromatic light obtained by coupling a white light source to a monochromator (Cornerstone CS260, Newport, USA) with a spectral band width of 10 nm. Fresh thylakoid membranes were used in each measurement, and the maximum photocurrent was recorded. This was repeated at different wavelengths, three times for each wavelength. The photon flux was obtained from the light intensity measured by a power meter (918D High Performance Photodiode Sensor, Newport, USA), and the EQE was calculated according to equation (3):


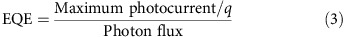


where *q* is the electron charge.

### Photoinhibition measurements

Photoinhibition experiments were done in the BPEC cell described above and with the same conditions as the photocurrent and hydrogen evolution measurements. Membranes were collected from the cell after 10 min incubation in the different conditions: dark/light, ±Fe(III)CN or ±Fe(II)CN. The thylakoids were re-suspended in fresh buffer to wash out the Fe(III)CN. Ten percent of the sample was taken for the measurement of the photosynthetic electron flow by spectroscopic analysis of the rate of DCMU and light dependent reduction of DCPIP (Sigma) serving as an indicator for oxygen production^42^, and the remaining 90% was used for photocurrent measurements. The photocurrent was measured using three electrode mode voltammetry (Ivium-n-Stat multichannel potentiostat, Ivium Technologies, The Netherlands) in fresh medium (buffer A, 3 mM Fe(III)CN, 0.09 mg Chl) under solar simulated illumination (model 10500 solar simulator, ABET Technologies, USA). DCPIP reduction was measured in 1 ml cuvette containing: buffer A, 50 μM DCPIP, thylakoids (10 μg Chl). The sample was illuminated with a white light halogen projector (0.15SU lamp, MRC). The DCPIP concentration was measured every 20 s with a monochromatic illumination at 600 nm for 100 s (using CO8000 cell density meter, WPA Biowave). The DCPIP reduction and the photocurrent values were normalized to the values measured when the membranes were placed in the BPEC cell (without Fe(III)CN) and immediately removed, centrifuged, re-suspended and divided 10% to DCPIP and 90% to photocurrent measurements.

### O_2_ evolution and consumption

For comparison with the photocurrent measurements the O_2_ evolution rate was measured in a Clark electrode (Hansatech Instruments, UK) with 0.1 mg Chl membranes and 3 mM Fe(III)CN as an electron acceptor. The sample was illuminated with a solar simulator (model 10500 solar simulator, ABET Technologies, USA) at 1 Sun for 10 min. For other measurements the O_2_ evolution rate was calculated for 0.1 mg Chl with 3 mM DCBQ, Fe(III)CN or 1,1′-dimethyl-4,4′-bipyridinium (MV, Aldrich) as electron acceptors with or without DCMU and DBMIB, under white light illumination (MRC). Reduced MV reduces the O_2_ and therefore it measures the consumption rate of O_2_.

### H_2_ evolution quantification

The H_2_ evolved at the cathode was collected into a designated glass tube, which was perforated at the bottom to allow buffer transfer, and sealed at the top with a rubber septum. Samples were collected from the headspace of the cell using a gas-tight syringe, and analysed by GC (7890A GC, Agilent Technologies, USA). The Faradaic efficiency for H_2_ generation was calculated from the ratio between the amount of H_2_ generated in the cell (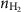, number of H_2_ moles generated during time interval *t*) and the amount of charge (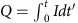) that was transferred between the working and counter electrodes during the same time interval (*t*) according to equation (4):


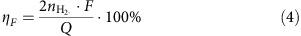


### Data availability

Data supporting the findings of this study are available within the article and its supplementary information file and from the corresponding author upon request.

## Additional information

**How to cite this article:** Pinhassi, R. I. *et al*. Hybrid bio-photo-electro-chemical cells for solar water splitting. *Nat. Commun.* 7:12552 doi: 10.1038/ncomms12552 (2016).

## Supplementary Material

Supplementary Information Supplementary Figure 1-6.
