# Supplementary material for: Hybrid bio-photo-electro-chemical cells for solar water splitting
Source: Nat Commun. 2016 Aug 23;7:12552. doi: 10.1038/ncomms12552 (PMC4996976; doi:10.1038/ncomms12552)
Supplement: Supplementary Information — Supplementary Figure 1-6. [file ncomms12552-s1.pdf]

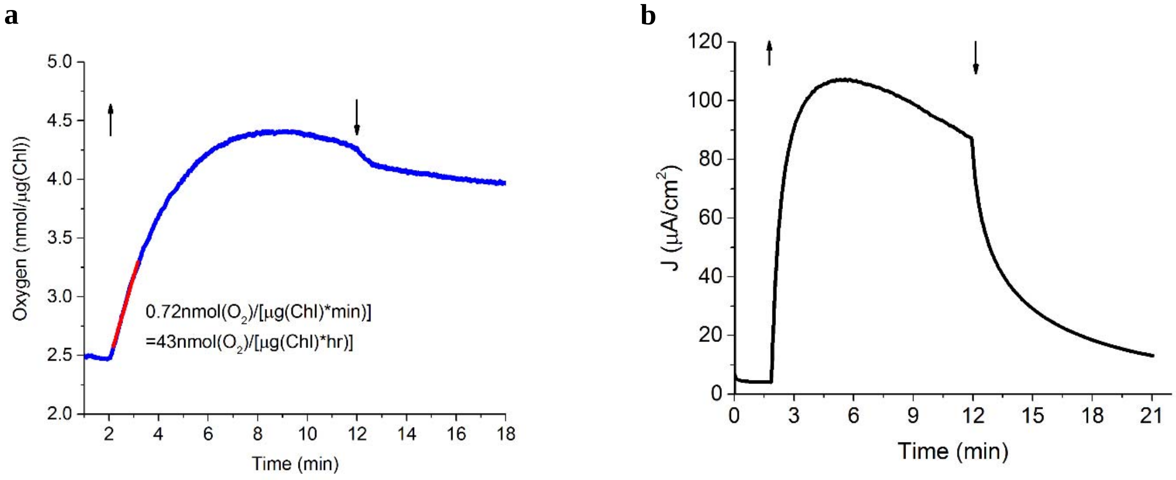

Supplementary Figure 1: Oxygen evolution to photocurrent efficiency from spinach thylakoids. (a) Oxygen evolution rate from spinach thylakoids. The measurements were done with a Clark electrode, in buffer A solution with a thylakoid content of 0.1 mg Chl and 3 mM Fe(III)CN, illuminated with a solar simulator at 1 Sun. The average (over 3 experiments) oxygen evolution rate was 43±3 µmol O_2 (mg Chl·h)$^{-1}$. This is lower than the oxygen evolution rate that was measured with 0.01 mg Chl, 190±12 µmol O_2 (mg Chl · h)$^{-1}$, due to the high Chl concentration. **(b)** Chronoamperometric measurements with 10 min of illumination. The total charge that was transferred was (94±11)% of the amount of oxygen evolved, averaged over 3

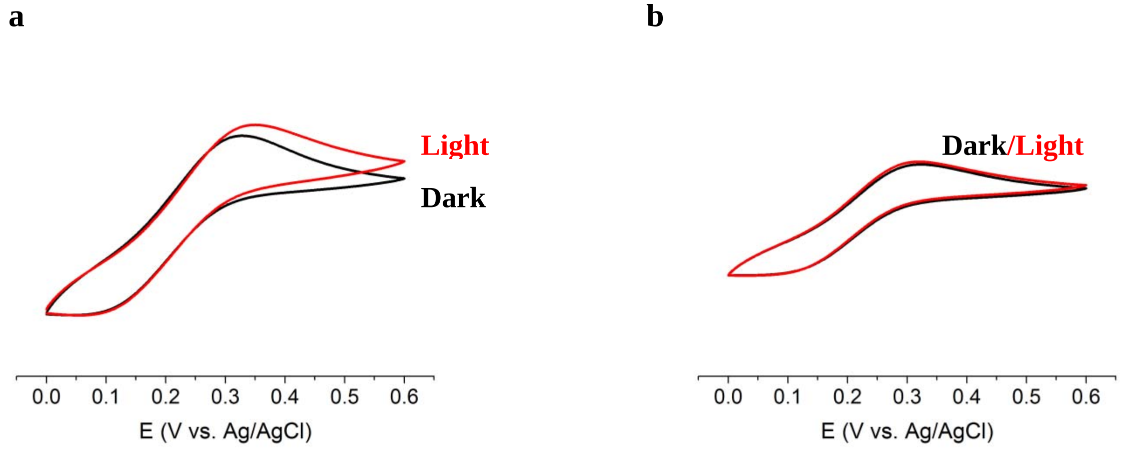

Supplementary Figure 2: The photocurrent is derived from photosynthetic electron transfer. (a) Cyclic voltammograms show photo-reduction current of Fe(III)CN at 300 mV vs. Ag/AgCl, indicated by the higher anodic peak in the light (red curve) compared to the dark (black curve). **(b)** DCMU (0.5 mM) inhibits the electron transfer from the thylakoids to Fe(III)CN, indicated by the absence of the photo-reduction current. The scan rate was 20 mV/s.

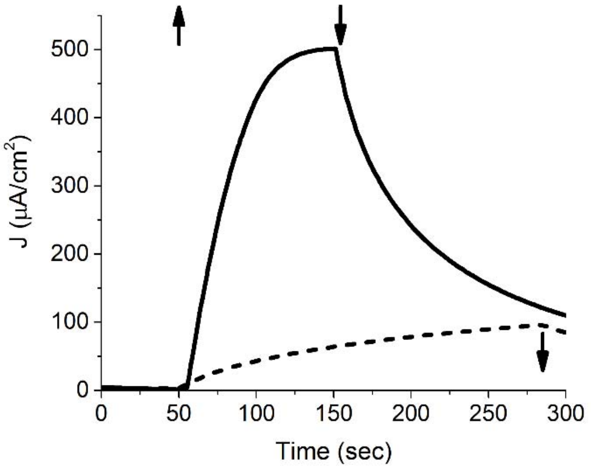

Supplementary Figure 3: BBY enriched membranes decreased the electron transfer to Fe(III)CN. Chronoamperograms measured at 0.5 V vs. Ag/AgCl with BBY (dashed line) and thylakoids (full line). Conditions were as described in the experimental methods with 0.1 mg Chl for both BBY and thylakoids, and with 3 mM Fe(III)CN. The BBY membranes produced much smaller photocurrent than the crude thylakoids, despite the fact that they gave rise to higher oxygen evolution rate (0.01 mg Chl to DCBQ). In this experiment the oxygen evolution rates were 386 ± 22 and 190 ± 12 μmol $O_2 \cdot (\text{mg Chl} \cdot \text{h})^{-1}$ for BBY and thylakoids, respectively.

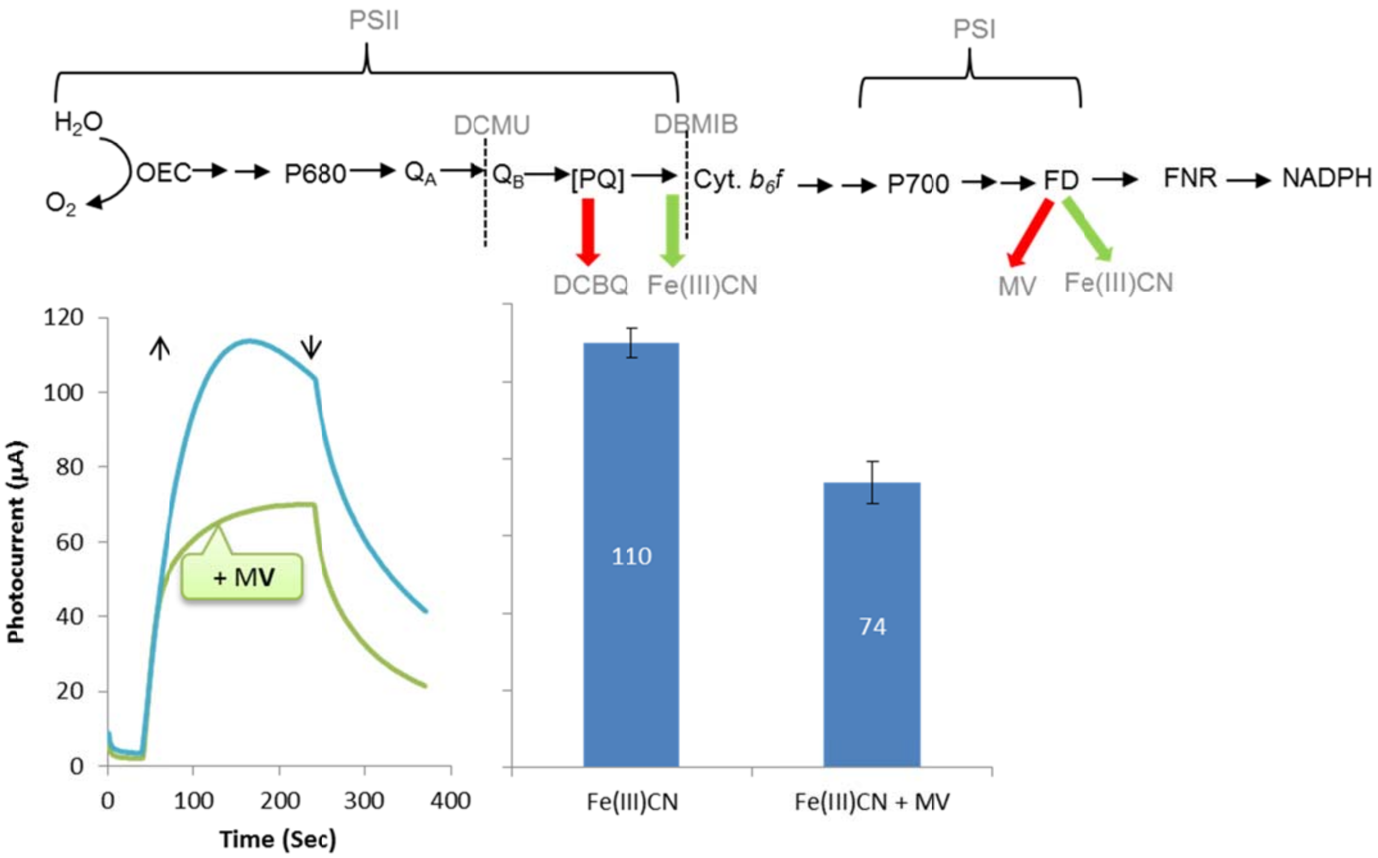

Supplementary Figure 4: Fe(III)CN is reduced by a component between Q_B and cyt $b_6 f$ and possibly also by ferredoxin (FD). Chronoamperometric measurements with (green line) or without (blue line) 3 mM MV. Conditions were as described in the experimental methods with 3 mM Fe(III)CN. Averaged over 3 measurements.

The results presented in Figure 4 of the article indicate that Fe(III)CN is reduced by PQ since the photocurrent was inhibited by DCMU but not by DBMIB. In order to analyze whether or not FD is also active in reducing Fe(III)CN in our system, MV was added and was found to inhibit the photocurrent by about 30%. MV competes with Fe(III)CN on the reduction activity of FD and therefore this result suggests that in addition to the reduction of Fe(III)CN by the first site located at the PQ, reduction of Fe(III)CN by FD also may contribute to the photocurrent (see scheme of the photosynthetic electron flow above). However, it should be noted that reduced MV produces oxygen radicals that could inhibit the photosynthetic electron flow and therefore reduce the photocurrent.

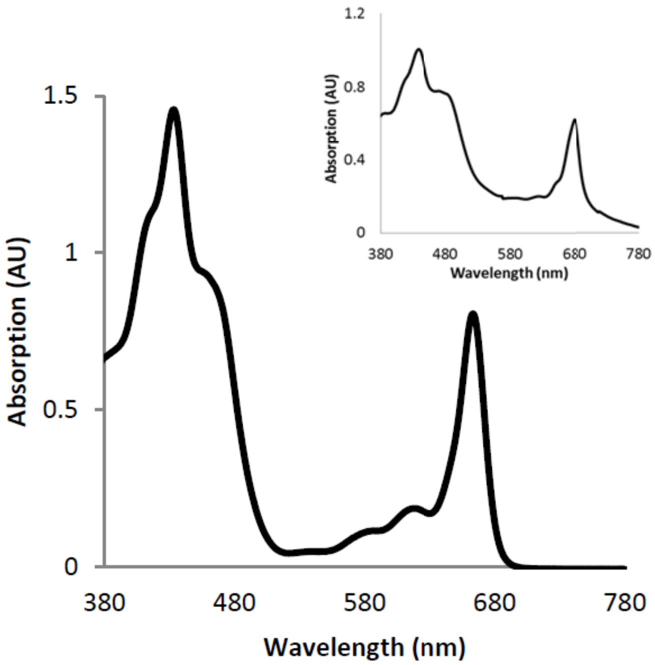

Supplementary Figure 5: Light absorption by chlorophyll from spinach thylakoids is maximal at wavelength 450 and 660 nm. Chlorophyll was extracted in 80% acetone. The inset shows a spectrum of chlorophyll from spinach thylakoids in buffer A solution.

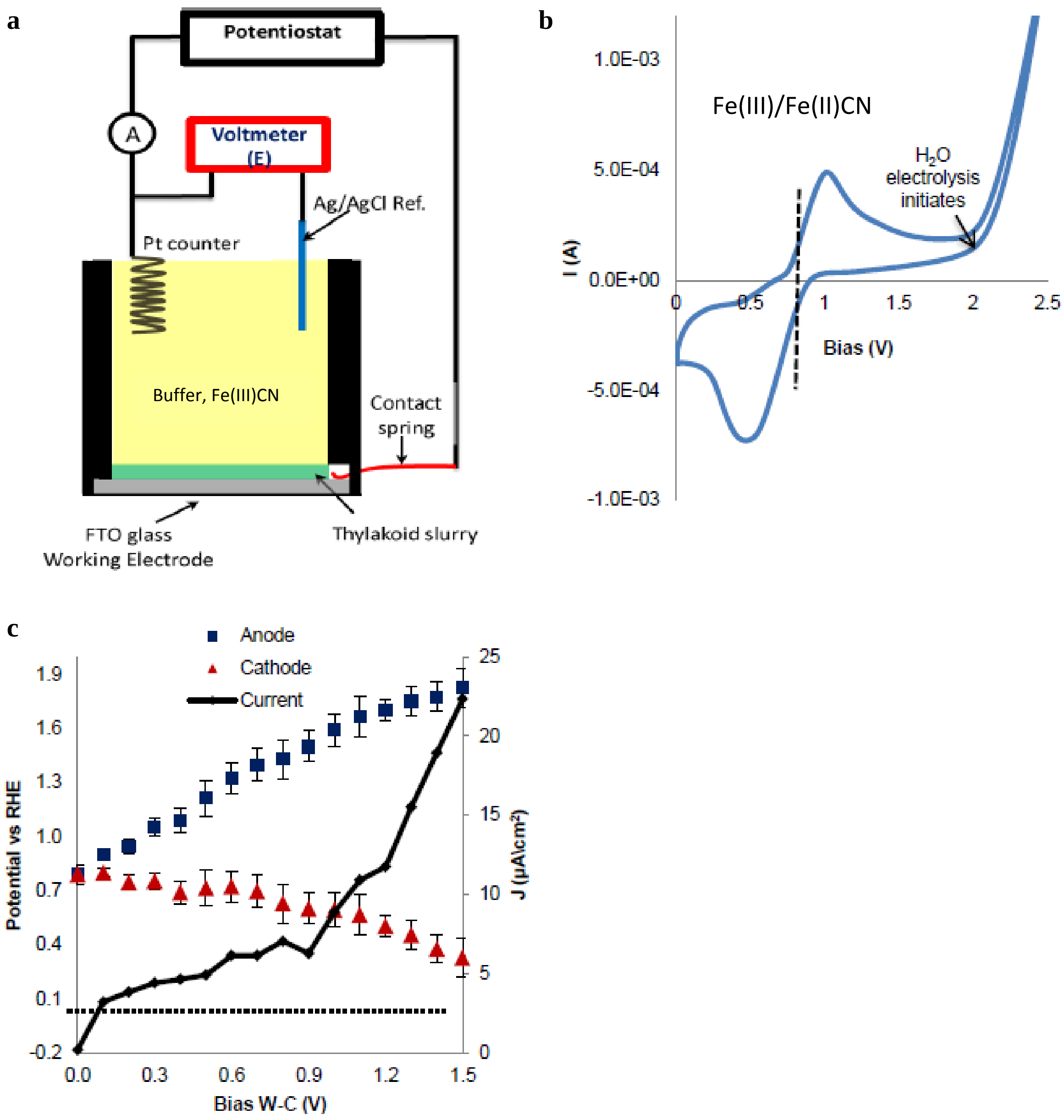

Supplementary Figure 6: **In the absence of thylakoid membranes water splitting initiates at a bias of 2 V. (a)** The setup used to evaluate the potential drop on the cathode in two-electrode mode. The Ag/AgCl reverence electrode is used here as a half cell with constant potential and assists to determine cathode voltage (V_{RHE}). **(b)** Cyclic voltammograms of the electrolyte solution containing 3 mM $K_3Fe(CN)_6$. The mid-point redox potential obtained is 0.78 V. Water electrolysis initiates at 2.0 V. **(c)** Control experiment where no thylakoids were present in the system. The voltage on the anode (blue squares) or on the cathode (red triangles) and the (dark) current produces by the photocell (black line) are presented as a function of the applied bias, measured under the setup presented in panel A. In the bias range 0-1.5 V the potential of the cathode was above 0 V_{RHE} (presented by a dashed line), which implies that hydrogen evolution could not have taken place.
